# New antibacterial candidates against *Acinetobacter baumannii* discovered by in silico-driven chemogenomics repurposing

**DOI:** 10.1371/journal.pone.0307913

**Published:** 2024-09-26

**Authors:** Kellen Christina Malheiros Borges, Vinícius Alexandre Fiaia Costa, Bruno Neves, André Kipnis, Ana Paula Junqueira-Kipnis

**Affiliations:** 1 Molecular Bacteriology Laboratory, Institute of Tropical Pathology and Public Health, Federal University of Goiás, Goiânia, Goiás, Brazil; 2 Microbiology Laboratory, Department of Biology, Academic Areas, Federal Institute of Goiás, Anápolis, Goiás, Brazil; 3 Laboratory of Cheminformatics, Faculty of Pharmacy, Federal University of Goiás, Goiânia, Goiás, Brazil; University of Buea, CAMEROON

## Abstract

*Acinetobacter baumannii* is a worldwide Gram-negative bacterium with a high resistance rate, responsible for a broad spectrum of hospital-acquired infections. A computational chemogenomics framework was applied to investigate the repurposing of approved drugs to target *A*. *baumannii*. This comprehensive approach involved compiling and preparing proteomic data, identifying homologous proteins in drug-target databases, evaluating the evolutionary conservation of targets, and conducting molecular docking studies and *in vitro* assays. Seven drugs were selected for experimental assays. Among them, tavaborole exhibited the most promising antimicrobial activity with a minimum inhibitory concentration (MIC) value of 2 μg/ml, potent activity against several clinically relevant strains, and robust efficacy against biofilms from multidrug-resistant strains at a concentration of 16 μg/ml. Molecular docking studies elucidated the binding modes of tavaborole in the editing and active domains of leucyl-tRNA synthetase, providing insights into its structural basis for antimicrobial activity. Tavaborole shows promise as an antimicrobial agent for combating *A*. *baumannii* infections and warrants further investigation in preclinical studies.

## Introduction

*Acinetobacter baumannii* stands out as a prominent pathogen accounting for a wide variety of severe infections affecting patients in intensive care units (ICUs), such as pneumonia, bacteremia, skin and soft tissue infections, wounds, urinary tract infections, and secondary meningitis [1]. Ventilator-associated pneumonia stands out among the most severe nosocomial infections attributable to multidrug-resistant (MDR), extensively drug-resistant (XDR), or pan drug-resistant (PDR; with resistance to all antibiotics available on the market) *A*. *baumannii* strains, closely followed by catheter-related bacteremia [2, 3].

The success of *A*. *baumannii* infection is due to several factors, such as the ability to form biofilms, resistance to desiccation on abiotic surfaces, ability to adhere, colonize, and invade human epithelial cells, potential to acquire genetic material through horizontal gene transfer, and to antimicrobial resistance mechanisms. The diverse and complex antibiotic resistance repertoire can occur through four main mechanisms: the presence of efflux pumps (perform drug extrusion), outer membrane protein modifications or the presence of capsule (make it difficult or prevent drug penetration), changes in the site of action (prevent drug binding), and enzymatic inhibition (cause enzymatic inactivation of drugs) [4]. Among them, the enzyme inhibition mechanism is the most prevalent and worrying is related to the production of a wide variety of β-lactamases, which make strains resistant to different β-lactam agents, in particular to carbapenems, considered first-line antimicrobials for the treatment of infections caused by *A*. *baumannii* [5].

Disordered use of antibiotics leads to increased selective pressures and consequently causes the emergence and dissemination of drug-resistant pathogens. Consequently, *A*. *baumannii* strains resistant to carbapenems (CRAB) were identified as one of the leading causes of infections associated with health care worldwide, particularly in ICUs [6]. These trends have forced the exploration of new antibiotic treatment approaches. According to the “Global Priority List of Antibiotic-Resistant Bacteria to Guide Research, Discovery, and Development of New Antibiotics” by the World Health Organization (WHO), the carbapenem-resistant Gram-negative pathogens: *Acinetobacter baumannii* together with *Pseudomonas aeruginosa* and *Enterobacteriaceae* are considered critical priority number 1 for research and development of new effective antimicrobials [7].

Identifying interactions between drugs and essential targets of biological systems represents an important process in drug discovery. Regarding the need to discover new antibacterial drugs, both the research and development (R&D) of these molecules are costly (approximately 2.8 billion dollars) [8], time-consuming (approximately 13.5 years) and bear high failure rates, since only 10% of drug candidates at the clinical stage are successfully launched in the market [9, 10]. Drug repurposing has emerged as a promising strategy to find new antibacterial candidates to help overcome these limitations [11]. Drug repurposing aims to identify new applications for drugs already approved or in the final stages of clinical trials [12]. This approach has several advantages over traditional drug discovery processes. Firstly, drug repurposing uses existing safety and toxicity data, and it significantly reduces both the time and cost required for preclinical and early clinical development processes. Secondly, drug repurposing can help find unexpected and innovative action mechanisms, broadening the current understanding of drug biology and potentially identifying new therapeutic applications [12]. Thus, drug repurposing could help reduce costs, risks, and timelines to the market; consequently, it could provide a strategic advantage in identifying new treatments for *A*. *baumannii* infections.

A growing collection of available computational and experimental methods that utilize molecular and clinical data enables diverse drug repositioning strategies. In this context, *in silico* chemogenomics approaches represent an effective strategy, as they compare the broad chemical spectrum of existing drugs with complete proteome and other potential drug targets. This approach helps identify compounds capable of acting on targets for which there is no known activity, but which have a homologous relationship with targets of active compounds already recognized [13, 14]. Molecular docking studies, in turn, represent a valuable approach for understanding the interaction between chemical compounds and their respective targets, through the exploration of ligand conformations in the binding sites of macromolecular targets. Docking also allows estimating the free energy of ligand-receptor binding, enabling the evaluation of critical phenomena involved in the intermolecular recognition process. In the context of drug repositioning, this tool can currently be used to predict new therapeutic indications for drugs with already optimized safety profiles, especially when used in addition to other computational methods, such as chemogenomics approaches [15]. Researchers willing to benefit from the wealth of genomic and proteomic information available for *A*. *baumannii* strains [16] can use computational chemogenomics tools to systematically identify potential drug candidates to fight this pathogen. This approach uses comprehensive genomic data to predict potential ligands capable of targeting specific proteins or pathways within *A*. *baumannii* based on the principle that proteins sharing enough similarity (homology) are more likely to share the same ligands [13, 17]. Current repurposing studies, based on computational chemogenomic strategies followed by experimental validation, generated promising results in the process of screening drugs showing activity against *Paracoccidiodes* spp. [17], *Plasmodium* spp. [18], *Histoplasma capsulatum* [19] and *Mycobacterium abscessus* [20]. These cutting-edge methods represent a significant leap forward in addressing the emergency posed by *A*. *baumannii* infections and hold great promise for future antimicrobial drug discoveries.

Considering the global threat of antimicrobial-resistant *A*. *baumannii* and their therapeutic challenges, this work aimed to carry out a computational chemogenomics approach to repurpose clinical trial-approved and candidate drugs; evaluate their *in vitro* activity against *A*. *baumannii*; select the most promising drug and analyze homology modeling and molecular docking interactions. The aforementioned approach comprised the following steps: (a) gathering and preparing *A*. *baumannii* genome data; (b) identifying homologous *A*. *baumannii* proteins in publicly available drug-target databases; (c) analyzing the evolutionary conservation level of prioritized *A*. *baumannii* targets, based on their phylogenetic association with homologs; and (d) conducting homology modeling and molecular docking studies. Tavaborole was prioritized for *in vitro* experimental validation against *A*. *baumannii* based on the designed pipeline. Antifungal tavaborole had minimum inhibitory concentration (MIC) values ranging from 2 to 64 μg/ml against both *A*. *baumannii* standard ATCC strain and MDR clinical isolates, as well as showed strong potential to reduce both the biomass and metabolic activity of biofilms formed by MDR clinical isolates at the concentration of 16 μg/ml.

## Materials and methods

### Computational chemogenomic analyses

#### Repurposing putative drugs available in public databases

Drug targets available in public databases were screened based on the assumption that homologous proteins are more likely to share the same ligands [13]. Sequence-based similarity search comprising *A*. *baumannii* proteins and all drug targets available on the Therapeutic Targets Database (TTD) [21] and DrugBank [22] was carried out based on using the OrthoVenn2 server [23]. A cut-off point of *≤* 10^−15^ for the expected value (*E*-value) was used to calculate pairwise sequence similarities among all input protein sequences. Subsequently, the sequence identity between *A*. *baumannii* proteins and their respective homologous targets was confirmed based on using the BLASTp server [24]. Sequence identity ≥ 30% was used as a cut-off point to prioritize *A*. *baumannii* proteins for subsequent analysis. Only paired *A*. *baumannii* proteins and drug targets with higher sequence identity or higher *E*-value were used for prospective investigations.

#### Data mining and computational prediction of essentiality

The datasets comprising homologous targets and corresponding drugs from DrugBank and TTD databases underwent merging and curation to eliminate redundancy. A stringent data mining procedure ensued to retain exclusively clinically approved or candidate drugs within the dataset. Drugs in experimental and investigative stages, as well as discontinued and illicit drugs, were excluded from the study. Finally, proteins associated with essential *A*. *baumannii* genes were identified and selected using the DEG database [25].

#### Comparing functional regions between drug targets and orthologs

FASTA sequences of potential *A*. *baumannii* protein targets were subjected to ConSurf server [26] to enable estimating the evolutionary conservation of amino acids, based on their phylogenetic association with homologs. Firstly, 150 homologous sequences were imported from the UNIREF-90 database [27] based on using an *E*-value cut-off of 10^−4^. Redundant sequences (identity > 95%) or sequences with minimal identity (< 35%) were ignored. Then, multiple sequence alignment (MSA) of homologous sequences was performed based on the MAFFT-L-INS-i method, whereas a phylogenetic tree was built by using neighbor-joining with maximum likelihood (ML) distance. Next, evolutionary conservation scores were calculated for position-specific amino acids, based on the empirical Bayesian method. Finally, local alignment was performed based on using the BLASTp server [24] to manually assess the sequence identity of conserved regions between *A*. *baumannii* proteins and their respective orthologous targets. Results were considered satisfactory when the conservation degree observed for active site residues was higher than, or equal, to 50% [13].

#### Rational selection of drugs

TTD, DrugBank, and PubChem platforms [21, 22, 28] were used to gather information on the compounds’ pharmacological properties and therapeutical indications of the selected drugs. Information about the molecular function of each target was collected in the UniProt database [29]. Metabolic pathways of the aforementioned targets were investigated in the Kegg Pathway database [30] and information about the subcellular location was collected in the Gram-LocEN database [31]. A literature search was carried out in the PubMed database [32] to identify approved drugs that had not yet been assessed against *A*. *baumannii* by querying all previously identified predicted drugs. Combinations of the following descriptors were used as a structured search strategy: (’’drug name’’ [MeSH Terms] OR ’’drug name’’ [All Fields]) AND (*’’Acinetobacter baumannii*’’ [MeSH Terms] OR ’’*Acinetobacter baumannii*’’ [All Fields]). Information about the chemical properties of both the investigated drugs and the biological properties of *A*. *baumannii* targets, which had been gathered at the previous research stage, was assessed for rational drug selection purposes. The following drug properties were analyzed: drug indication, absorption, distribution volume, protein binding, metabolism, excretion route, half-life, clearance, log P, log S, and toxicity. As for *A*. *baumannii* targets, the following parameters were assessed: molecular function, biological process, metabolic pathway, and subcellular location. Thus, some drugs at the clinical trial stage were ruled out because they were not available for purchase. Moreover, vaccines, nutraceutical compounds, coenzymes, genetic engineering vectors, and all compounds lacking information about their pharmacological properties, such as absorption, distribution, metabolism, excretion, and toxicity, were also discarded.

### Molecular modeling

#### Target 3D structure prediction

FASTA sequence of *A*. *baumannii* leucyl-tRNA synthetase (LeuRS) (UniProt code: A0A7Z1WR47) was used as an input file for 3D structure prediction purposes, based on the following approaches: (*i*) homology modeling implemented in SWISS-MODEL server [33], (*ii*) threading method available in I-TASSER server [34], and (*iii*) *ab initio* method based on graph attention neural network available in AlphaFold server [35]. The 3D structure of *Thermus thermophilus* LeuRS (PDB ID: 2V0C, sequence identity: 46.88%) was used as a template for SWISS-MODEL prediction [36].

#### Geometric analysis of LeuRS 3D structure

The 3D geometric quality of the LeuRS model was investigated using the MolProbity server [37]. 3D structures presenting the most favorable Ramachandran angles (phi [φ] and psi [ψ]) within the preferred regions, and the ones recording the lowest Clashscore and MolProbity score, were selected for further analysis.

#### Tavaborole binding site identification

The LeuRS model was subjected to the ConSurf server [26] to estimate the evolutionary conservation of amino acids based on their phylogenetic association with homologs [38] using the abovementioned parameters. At the same time, computational fragment mapping calculations were performed based on using the FTMap server [39] to identify small molecules binding hot spots in both the connective polypeptide 1 (CP1) amino acid editing domain and LeuRS’s active site. FTMap can detect potential binding sites by using 16 small organic molecules with different shapes and polarities in a dense grid around the LeuRS structure.

#### Ligand and protein preparation

The 2D structure of tavaborole was imported into the Maestro workspace v.9.3 (Schrödinger, LCC, New York, 2012). Subsequently, the 3D structure of tavaborole underwent meticulous optimization using the OPLS_2005 force field [40], a feature available on LigPrep v.2.5 (Schrödinger, LCC, New York, 2012). Subsequently, tavaborole’s 3D structure was imported to AcePrep, where it underwent preparation steps comprising counterions’ removal, protonation, and generation of 10 conformers. Simultaneously, LeuRS’s predicted 3D structure was prepared using the ProteinPrepare web application. Protonation state titration was calculated at pH 7.4, in PROPKA software, v.3.1, missing atoms were added, and this procedure was followed by overall hydrogen network optimization in PDB2PQR software, version 2.1.

#### Molecular docking

Molecular docking studies were carried out at LeuRS’s predicted binding sites in rDock software implemented in the AceDock server [41]. The analysis was conducted through a cross-docking approach, which comprises the application of ligand structures obtained from multiple PDB files of the same protein in comparison to a single rigid protein model structure. This process was performed by using the free docking mode and the pharmacophore-overlapping strategy [41]. The template ligand in the free docking mode was only used to establish the search space for the docking run; pose prediction was conducted by taking into consideration the rDock master scoring function. On the other hand, in pharmacophore-overlapping re-scoring, the predicted poses were assessed based on how well their pharmacophoric moieties (such as aromatic rings, and hydrogen bond donors, among others) align with those of the template ligand. The robustness of the docking protocol was assessed by calculating the enrichment rates (see details in S3 File) and root mean squared deviation (RMSD) between the docked pose and the corresponding crystal conformer [42, 43]. RMSD values lower than 2.0 Å were considered satisfactory, whereas values close to zero were considered optimal.

#### MM/GBSA free energy calculation

A combination of molecular docking simulation protocols and MM/GBSA (Molecular Mechanics-Generalized Born Surface Area) methodology were employed to investigate the interaction between tavaborole and LeuRS [44]. The MM/GBSA approach was utilized, incorporating computed molecular mechanics energies and implicit solvation models to determine the energy difference between the bound complexes (tavaborole-LeuRS). The estimation of relative binding affinity used the MM/GBSA module within the Prime Module of the Schrödinger suite, employing the OPLS_2005 force field and VSGB solvation mode. Initially, the software initiates the process by minimizing the receptor-ligand complex and then calculates energies for each ligand, receptor, and combined complex. Subsequently, the ΔG binding energy is computed using the following equation:

$${\Delta G}_{binding}={\Delta G}_{tavaborole-LeuRS}-\;{\Delta G}_{tavaborole}-\;{\Delta G}_{LeuRS}$$

The MM/GBSA calculates the approximate binding energy of a receptor-ligand complex. A greater negative value indicates a more robust binding between the specified ligand and receptor [44].

### Experimental procedures *in vitro*

#### Chemicals

All compounds were ≥ 95% pure, solubilized in dimethyl sulfoxide (DMSO), and stored as stock solutions at the concentration of 10 mg/ml. The following drugs prioritized for experimental validation purposes were purchased from ChemScene:

1. atovaquone, CAS Registry Number (CAS n.) 95233-18-4, molecular weight (MW) 366.84;
2. homoharringtonine, CAS n. 26833-87-4, MW 545.62;
3. leflunomide, CAS n.75706-12-6, MW 270.21;
4. MKT-077 (1-Ethyl-2-[[3-ethyl-5-(3-methyl-2(3H)-benzothiazolylidene)-4-oxo-2-thiazolidinylidene]methyl]-pyridinium chloride), CAS n. 147366-41-4, MW 432.00;
5. ribavirin, CAS n. 36791-04-5, MW 244.20;
6. tavaborole, CAS n.174671-46-6, MW 151.93;
7. thiabendazole, CAS n.148-79-8, MW 201.25.

#### Microorganisms and culture conditions

All microbial clinical strains were derived from the Brazilian Culture Collection of the Tropical Pathology and Public Health Institute—BCC-IPTSP (https://rgptb.iptsp.ufg.br/). Clinical strains’ resistance profiles and biofilm formation ability were described by Castilho et al. [45]. In addition, a standard *A*. *baumannii* sample from the American Type Culture Collection (ATCC 19606) was used in this study. Isolates were reactivated in Luria Bertani (LB) agar (Kasvi) and incubated in a bacteriological incubator at 36°C, for 18 hours. Colonies isolated from each reactivated strain were used to mount microscope slides and stained by the Gram technique to assess both morphological features and purity of each bacterial isolate. All strains belonging to each isolated colony were inoculated in LB broth after their purity and viability were confirmed. After growing in an orbital shaking incubator at 36°C for 24h, strains were prepared for storage and working-aliquot maintenance. They were kept in 20% glycerol, in a freezer, at -20°C, to preserve the bacterial isolates used in the microbiological assays.

#### Determining the minimum inhibitory concentration (MIC)

The broth microdilution method was applied to estimate candidate drugs’ MIC by following standards set by the Clinical and Laboratory Standards Institute (CLSI) [46], with small modifications. Cultures were seeded in LB broth added with 0.05% Tween 80 and incubated in an orbital shaking incubator at 36°C, for 24h. After this period, bacterial cultures had turbidity assessed in a spectrophotometer (Kasuaki IL-226 Spectrophotometer, Jiangsu, China) at 590 nm; culture concentration was adjusted to 2x10^6^ CFU/ml in LB. The antibiotic amikacin (Sigma-Aldrich, St. Louis, MO, USA) was used at 8 μg/ml as a positive inhibition control [CAMI]. Drugs’ antimicrobial activity was assessed at the following concentrations: 1,024; 512; 256; 128; 64; 32; 16; 8; 4; 2; 1; 0.5; 0.25; 0.125 and 0.0625 μg/ml. After preparation, the plate was incubated at 36°C, for 24h. After the incubation period, 30 μl of resazurin solution (0.01%) was added to each well, according to the resazurin-based 96-well plate microdilution method, to determine MIC, with adaptations [47]. The plate was incubated again, at 36°C, and observed every 10 minutes to check color changes in the growth control wells [C+]. The MIC was determined as the lowest drug concentration at which resazurin wells remained blue through visual reading. Assays focused on assessing the antimicrobial potential of each drug were performed in triplicate and repeated at different time intervals.

#### MKT-077 antimicrobial susceptibility test

MKT-077 is a synthetic heterocyclic pyridinium compound analogous to rhodacyanine dye; its dark orange color visually and colorimetrically affects experimental assays. Therefore, the serial microdilution method was used to assess the sensitivity degree of the analyzed strain, and it was followed by plating each serial drug dilution into Petri dishes covered with LB agar medium to find the minimum bactericidal concentration (MBC) [46]. A culture microplate was prepared and incubated in compliance with previously described procedures for other drugs, at the same concentration range: 1,024 to 16 μg/ml. After the incubation process at 36ºC for 24h was over, each culture microplate well was serially diluted at a scale of 10 (ranging from 10^−1^ to 10^−6^); then, 10 μl of dilutions ranging from 10^−3^ to 10^−6^ were seeded on Petri dishes added with LB agar. Aliquots of 10 μl extracted from [C–] and [C^AMI^] wells without serial dilution were also seeded on a Petri dish added with LB agar. All Petri dishes were incubated in a bacteriological incubator at 36°C for 24h. After incubation, MBC was determined as the lowest MKT-077 concentration that fully inhibited bacterial growth.

#### Analyzing bacterial biofilm formation and mature biofilm inhibition

The estimated quantification of bacterial biofilm formation by clinical *A*. *baumannii* strains was performed in a microplate, based on the methodology described by Castilho et al. [45]. Cultures grown in LB broth were adjusted to the concentration of 2x10^6^ CFU/ml; 100 μl of this suspension was added to 100 μl of LB broth—1/4 of its concentration added with glucose at 0.2% (final volume of 200 μl per well)—and incubated at 28ºC, for 48h. Bacterial growth was measured in a microplate spectrophotometer (Multiskan SkyHigh Microplate Spectrophotometer, Thermo Fisher Scientific, Waltham, MA, EUA), at 405 nm. Then, the supernatant was removed and wells were washed twice with 200 μl of PBS. The attached biofilm was stained with 0.2% crystal violet, washed again, and, subsequently, solubilized with ethanol/acetone at the ratio of 80:20 (v/v).

Crystal violet staining assays were carried out to assess biofilm mass, and resazurin assays were used to estimate viable counts, to investigate likely tavaborole activity on mature biofilm. Previously formed biofilms were washed, as described above, and serially diluted tavaborole was added to them at final concentrations ranging from 2 to 16 μg/ml; then, the plate was incubated again for 24h. After the incubation, the supernatant was discarded and the wells were washed. The biofilm was stained with 0.2% crystal violet solution, again washed, and, subsequently, solubilized with 200 μl of ethanol/acetone at the ratio of 80:20 (v/v); absorbance was measured (OD 595 nm) and biomass reduction rate was calculated based on Shen et al. [48]. In total, 45 μl of resazurin solution (0.01%) was added to each test and control well to check mature biofilm cell viability after drug administration. The plate was incubated at 36°C and observed every 10 minutes to investigate resazurin reduction to resorufin; then, the reduction rate was calculated by measuring absorbance at 570 and 600 nm. Metabolic activity reduction rate was calculated based on das Neves et al. [47].

#### Statistical analysis

All assays were performed in triplicate and repeated at different intervals under each tested condition. Data deriving from tests conducted *in vitro* were inserted in an Excel spreadsheet. Both mean and standard deviation (SD) within samples were calculated. GraphPad Prism Software (GraphPad Software Inc., San Diego, CA, USA) was used for data analysis and graphical representation.

## Results

### Computational chemogenomic analyses

**Fig 1:**
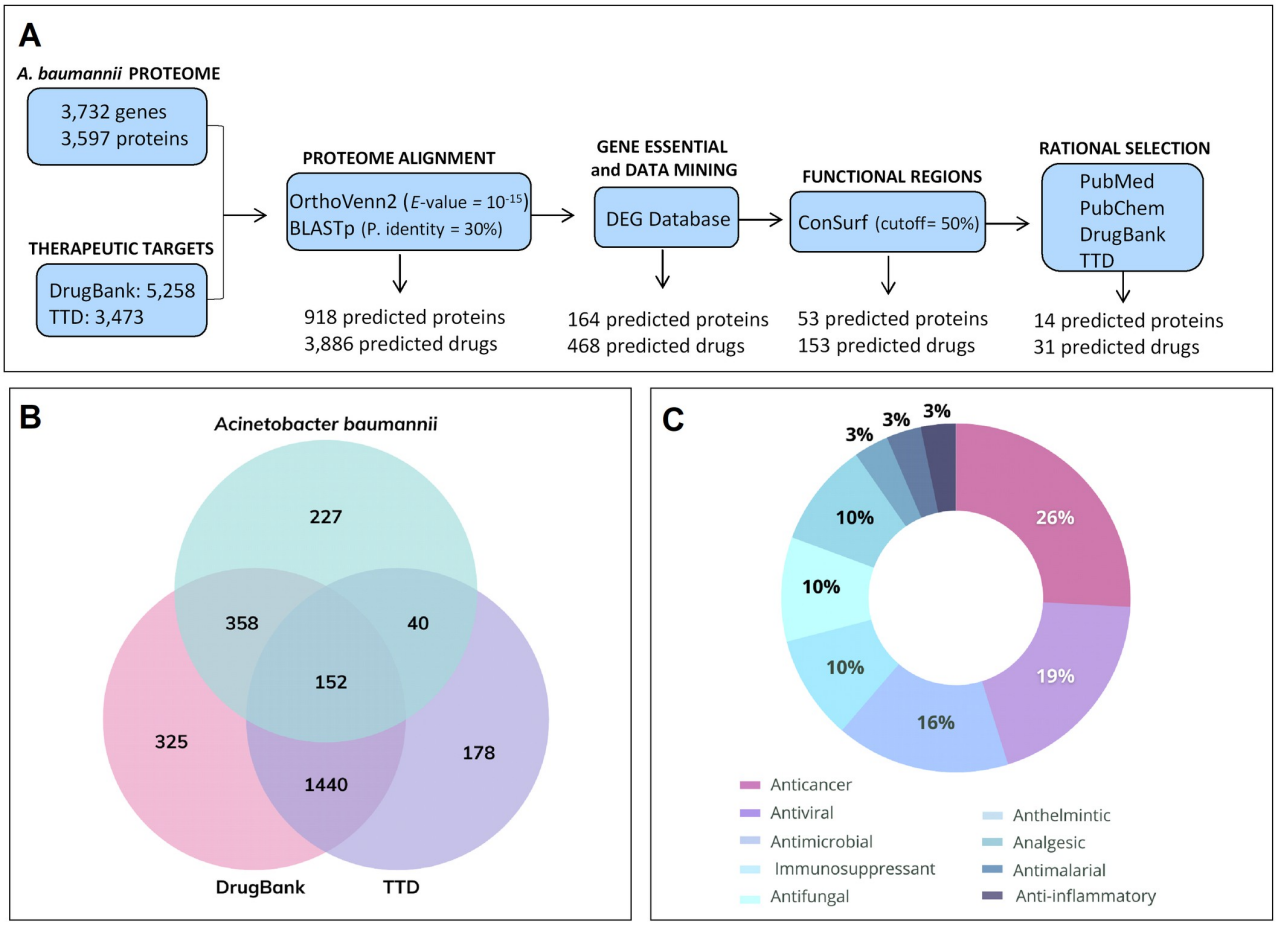
Computational chemogenomic analyses. (A) Workflow diagram showing the computational steps and the number of drugs screened in each step. (B) Venn Diagram. Clusters generated by comparative analysis between therapeutic targets deriving from DrugBank (510 clusters comprising 1,402 orthologs) and Therapeutic Target (TTD) (192 clusters comprising 676 orthologs) databases and proteins deriving from *Acinetobacter baumannii*. (C) Therapeutic recommendation of 31 predicted drugs based on computational analysis.

A computational chemogenomics framework was developed (Fig 1A) to repurpose drugs to treat bacterial infections based on predicted protein analysis applied to 3,732 genes (3,597 proteins) from *A*. *baumannii* genome (Genome Assembly ASM863263v1). Then, each one of these proteins was used to investigate two different publicly available databases, DrugBank and TTD, which provide detailed information about drugs and their targets. This strategy identified 918 potential bacterial targets (∼25.6% of the investigated *A*. *baumannii* targets; DrugBank: 1,402 orthologs; TTD: 676 orthologs) (Fig 1B) that might interact with 3,886 ligands.

Subsequently, DrugBank and TTD were merged for manual data mining and duplicate removal purposes. In total, 483 protein targets were maintained in this phase; they corresponded to 1,995 drugs (1,392 from DrugBank and 603 from TTD). After additional data mining (to select approved drugs or drug candidates in clinical trials) and manual filtering (to keep only one ortholog protein per cluster) procedures were over, the information provided by DEG was used to both identify and select proteins associated with essential *A*. *baumannii* genes. In total, 164 protein targets were retained at the end of these selection processes; this number corresponded to 468 predicted drugs (403 from DrugBank and 65 from TTD).

ConSurf server was used to assess the active site conservation level among targets and determine the functional amino acids of drug targets. Then, local alignment was performed using the BLASTp server to compare *A*. *baumannii* protein sequences to therapeutic targets’ sequences (orthologs). In total, 53 protein targets were selected at the end of this analysis; they corresponded to 153 drugs (101 from DrugBank and 52 from TTD).

A search was conducted in the PubMed database to rule out previously assessed drugs showing antimicrobial activity against *A*. *baumannii*. Sixty known antibiotics were identified and excluded, including eleven currently used for treating *A*. *baumannii* clinical infections, such as amikacin, ampicillin, cefepime, and tigecycline among others. In total, 93 drugs (51 from DrugBank and 42 from TTD) targeting 33 proteins remained. The pharmacological properties and therapeutic indication of these drugs were analyzed through data collected from the TTD, DrugBank, and PubChem platforms. In total, 31 drugs (7 from DrugBank and 24 from TTD) were prioritized corresponding to 14 protein targets. Most of the drugs selected at this stage were previously featured as having anticancer activity (26%); they were followed by antivirals (19%), antimicrobials (16%), immunosuppressants (10%), antifungals (10%) and anthelmintics (10%) (Fig 1C).

Among all 31 selected drugs, seven were chosen for experimental analysis focused on investigating their potential use for repurposing against *A*. *baumannii*, based on previous analysis applied to homology rate, chemical structure diversity, pharmacological properties, and predicted target types. Thus, six FDA-approved drugs, atovaquone (*A*. *baumannii* predicted target: dihydroorotate dehydrogenase), homoharringtonine (50S ribosomal protein), leflunomide (dihydroorotate dehydrogenase), ribavirin (inosine-5’-monophosphate dehydrogenase), tavaborole (leucyl-tRNA synthetase), and thiabendazole (succinate dehydrogenase), and one drug undergoing clinical studies, MKT-077 (DnaK protein chaperone), were herein selected (Table 1). Two drugs belonged to the anticancer therapeutic class (MKT-077 and homoharringtonine), one drug had antifungal (tavaborole) activity, one had antifungal and antiprotozoal activity (atovaquone), one was originally indicated as an immunosuppressant (leflunomide), one had antiviral activity (ribavirin) and one was used in classic therapy as an anthelmintic drug (thiabendazole). The pharmacokinetic properties of drugs obtained from the DrugBank, TTD, and PubChem databases are described in S1 Table.

**Table 1:** Drugs predicted to act in *Acinetobacter baumannii* proteins and selected to be tested against strain ATCC 19606.

Drug	Original Indication	Original Target	*A*. *baumannii* Predicted Target/ UniProt ID	Evolutionary Conservation Rate (%)^a^	MIC ^b^	
					μg/ml	μM
**Tavaborole**	Antifungal	Leucyl-tRNA synthetase	Leucyl-tRNA synthetase (A0A7Z1WR47)	59.0	2	13.2
**Ribavirin**	Antiviral	Inosine-5’-monophosphate dehydrogenase; RNA-directed RNA polymerase	Inosine-5’-monophosphate dehydrogenase (P31002)	59.0	4	16.4
**Leflunomide**	Immunosuppressant	Dihydroorotate dehydrogenase	Dihydroorotate dehydrogenase (B0V824)	67.0	64	236.8
**Atovaquone**	Antiprotozoal	Dihydroorotate dehydrogenase	Dihydroorotate dehydrogenase (B0V824)	67.0	256	697.9
**Homoharringtonine**	Anticancer	50S ribosomal protein L2; 60S ribosomal protein L3	50S ribosomal protein L2 (B0V6X1)	58.0	256	469.2
**Thiabendazole**	Anthelmintic	Fumarate reductase flavoprotein subunit; Succinate dehydrogenase; Cytochrome P450	Succinate dehydrogenase (A0A2S4TCK3)	51.0	512	2544.1
**MKT-077**	Anticancer	Heat shock protein 70 (HSP 70)	DnaK protein chaperone (A3M8W9)	93.6	1024 ^c^	2370.4

Note:

^a^ Rate of shared conserved residues.

^b^ Minimum Inhibitory Concentration.

^c^ Minimum Bactericidal Concentration.

### Experimental assessment of selected drugs

Growth inhibition assays were conducted with the ATCC 19606 standard strain, at drug concentrations ranging from 1,024 to 0.0625 μg/ml, to assess the antimicrobial potential of drugs selected in the computational steps. MIC values observed for drug candidates are shown in Table 1. Among tested drugs, the antifungal tavaborole presented the most promising activity, with a MIC value of 2 μg/ml (13.2 μM). Ribavirin presented a MIC value of 4 μg/ml, whereas leflunomide showed a MIC value of 64 μg/ml. Both atovaquone and homoharringtonine recorded MIC values of 256 μg/ml. Thiabendazole, in turn, had a MIC value of 512 μg/ml against the investigated microbial strain.

Because tavaborole presented the best antimicrobial activity against the *A*. *baumannii* standard strain, among the herein-assessed drugs, the option was made to carry out a deeper analysis. Clinical isolates with different resistance profiles [45] were selected to assess tavaborole’s antimicrobial potential against them. Table 2 presents MIC values recorded for standard strain ATCC 19606 and for all herein assessed 14 clinical strains (MIC values ranged from 2 to 64 μg/ml, with MIC_50_ = 2 μg/ml and MIC_90_ = 32 μg/ml).

**Table 2:** Tavaborole’s minimum inhibitory concentration (MIC, g/ml) against ATCC 19606 strain and 14 MDR *Acinetobacter baumannii* clinical isolates.

Strain (n = 15)	MIC (μg/ml)
ATCC 19606	2
Ab23	2
Ab33	2
Ab35	2
Ab38	16
Ab40	64
Ab50	2
Ab53	2
Ab54	32
Ab59	2
Ab61	2
Ab62	2
Ab71	16
Ab72	4
Ab88	4

To investigate the antimicrobial potential of MKT-077, which is dark orange, MBC was determined by testing this drug at concentrations ranging from 1,024 to 16 μg/ml. MBC recorded for the assessed standard strain was 1,024 μg/ml (S1 Fig).

**Fig 2:**
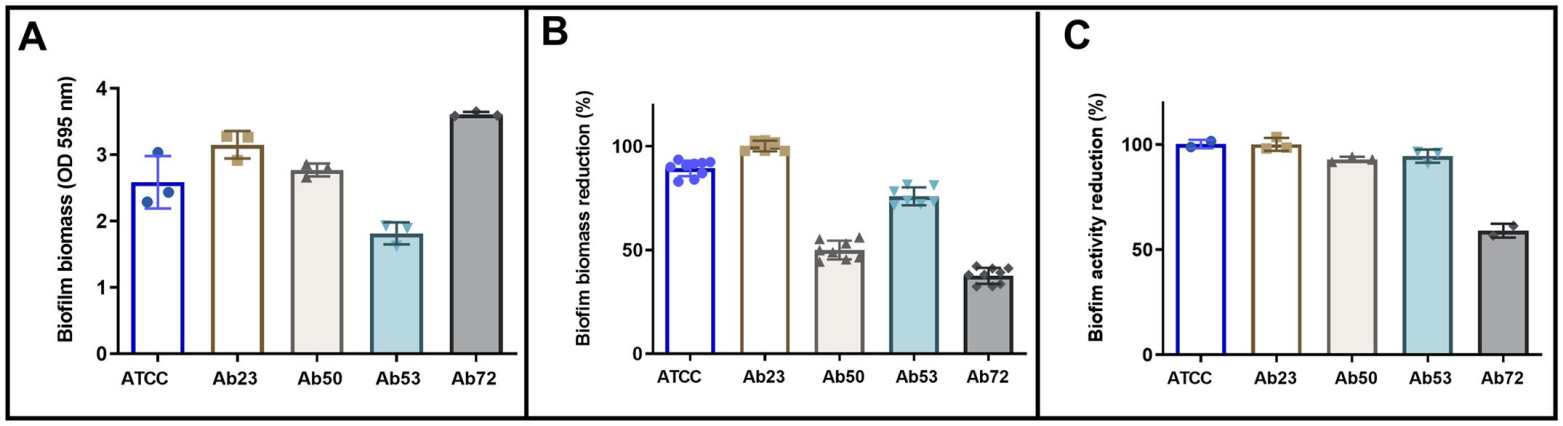
Biofilm formation and tavaborole effect on mature biofilm of five *Acinetobacter baumannii* strains in polystyrene plates. (A) Quantification of biofilm biomass formed after 48h incubation without the presence of the investigated drug determined through OD reading at 595 nm. Each symbol represents the mean of triplicates. (B) Percentage of OD reduction for biofilm biomass treated with 16 μg/ml tavaborole for 24h. (C) Metabolic activity reduction rate recorded for biofilm treated with 16 μg/ml tavaborole for 24h. The bars represent the mean and standard deviation of different experiments. Three independent experiments were performed.

Because tavaborole also presented antibacterial activity against clinical *A*. *baumannii* isolates when it was assessed in its planktonic form, an option was made to investigate its activity against mature biofilm, at concentrations ranging from 2 to 16 μg/ml. The investigated *A*. *baumannii* strains produced relatively similar biofilm amounts, with emphasis on the higher production of it in the Ab72 strain (Fig 2A). Tavaborole, at the concentration of 16 μg/ml, significantly reduced mature biofilm amount in Ab23 (100%), ATCC (89%) and Ab53 (76%) isolates, as well as slightly reduced it in Ab50 (50%) and Ab72 isolates (38%) (Fig 2B). A resazurin test was carried out to confirm viable bacteria reduction. Results have shown that this drug, at the concentration of 16 μg/ml, reduced ATCC (100%), Ab23 (100%), Ab50 (93%), Ab53 (94%), and Ab72 (59%) viabilities (Fig 2C).

### Structural basis for tavaborole antimicrobial activity

Tavaborole was chosen for further investigation as it showed the best antimicrobial activities against *A*. *baumannii*. It is predicted to target LeuRS of *A*. *baumannii* due to its high evolutionary conservation with LeuRS of fungi. As shown in Table 1, the LeuRS of *A*. *baumannii* shares a 59% conservation rate with fungal LeuRS, which tavaborole is known to inhibit effectively. This conservation suggests that a similar mechanism of inhibition could be leveraged in *A*. *baumannii*. LeuRS has been identified as a potential target for antibacterial agents due to its critical role in protein synthesis and its evolutionary conservation across different bacterial species. Inhibitors of LeuRS, such as tavaborole, can disrupt this process, inhibiting bacterial growth, and making it a promising target for developing new antibacterial therapies [49]. Given this, a molecular docking study was conducted with LeuRS deriving from *A*. *baumannii* to explore the structural basis of tavaborole’s antibacterial activity. Since LeuRS’s 3D structure was not available in the PDB when the current study was conducted, its 3D structure was computationally predicted using SWISS-MODEL, I-TASSER, and AlphaFold structures. Then, predicted 3D structures (S1 and S2 Files) were geometrically optimized and their structural quality was ensured using different structural description levels. Details of geometric features of predicted LeuRS structures are described, in detail, in S2 Table. The geometric analysis applied to 3D models evidenced that most amino acids fell within the favored Ramachandran regions (88.42% − 97.59%); this finding indicated the high quality of torsional angles φ and ψ of N-Cα and Cα-C bonds. These models also presented acceptable Clash (ranging from 0.95 to 3.29) and MolProbity scores (ranging from 0.87 to 2.50).

**Fig 3:**
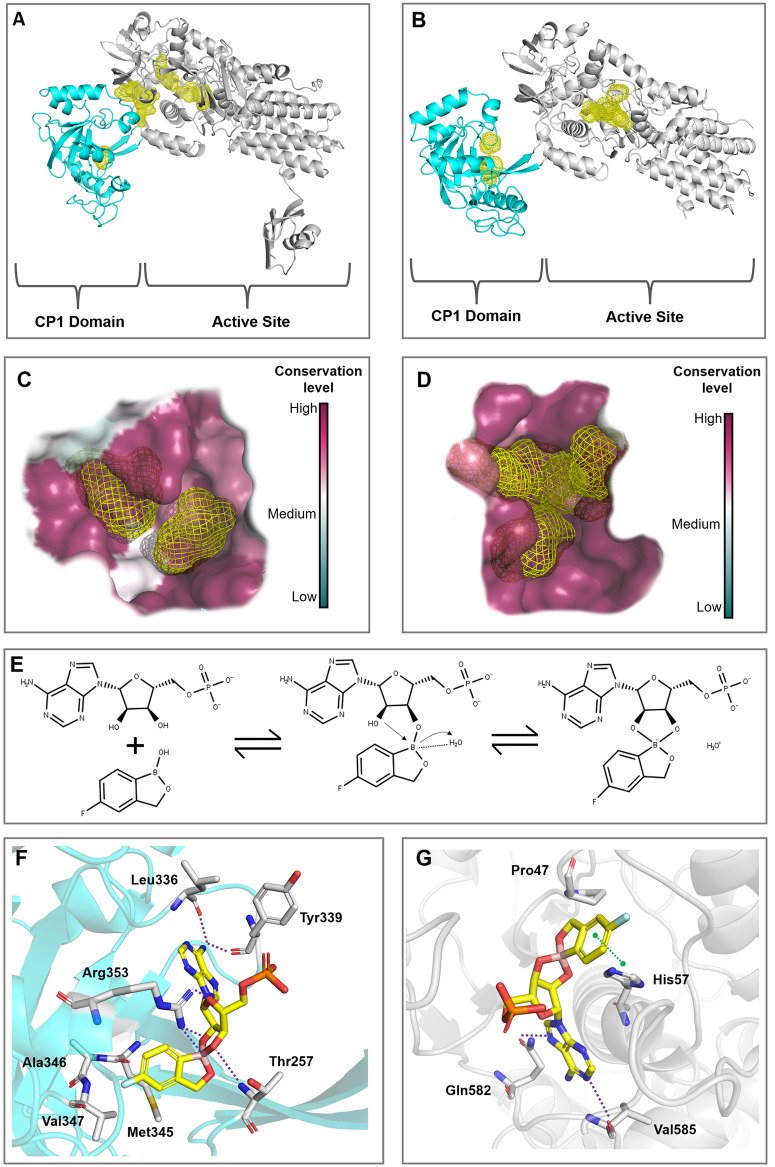
Leucyl-tRNA synthetase structural analysis and molecular docking. (A) Predicted hot spots (yellow) in the AlphaFold structure. (B) Predicted hot spots (yellow) in the SWISS-MODEL structure. LeuRS’s CP1 and active site domains were highlighted in cyan and gray, respectively. (C) and (D) show the evolutionary conservation level of amino acids around the hot spots in the CP1 domain and in the active site, respectively. (E) Likely tavaborole-inhibition mechanism in LeuRS’s CP1 domain. The boron atom found in tavaborole triggers the attack on the 2’OH of the AMP ribose. Benzoxaborole, at the LeuRS CP1 domain, forms a covalent adduct with AMP by using its boron atom to bind with adenosine 2’ and 3’ oxygen atoms. The boron atom carries a negative charge within the tRNA-AN2690 adduct and achieves stabilization by interacting with a protonated water molecule. (F) and (G) represent tavaborole’s predicted binding modes in LeuRS’ CP1 domain and active site, respectively. Residues interacting with the ligand are shown in gray sticks, whereas the π-π stacking and hydrogen bonds are highlighted in green- and purple-dashed lines, respectively.

Hot spot analysis was conducted with the best-predicted AlphaFold and SWISS-MODEL structures to identify energetically favorable binding pockets within LeuRS; these structures were used because they present different conformations. Fig 3A and 3B show the most promising binding regions, highlighted with yellow surfaces to increase the visibility of these hotspots, and highlight the main conformational differences in the CP1 domain and the active site of the structures predicted by SWISS-MODEL and AlphaFold, respectively. These differences are essential to help identify cryptic sites and assess the conformation impact on tavaborole’s binding mode. According to the hot spots analysis (Fig 3A and 3B), the AlphaFold structure has shown two potential small-molecule binding sites in the CP1 domain and active site, respectively. Moreover, a hot spot cluster was observed between the active site and the CP1 domain. On the other hand, the SWISS-MODEL structure presented two potential binding sites within the CP1 domain and the active site, even though it did not show a cryptic site at the domain interface.

Subsequently, ConSurf analysis was used to estimate the conservation degree of LeuRS amino acids and, consequently, to predict the biological significance of predicted binding sites, since functionally important regions tend to be more evolutionarily conserved than other positions. As shown in Fig 3C and 3D, predicted binding pockets in the active site and CP1 domain (SWISS-MODEL) presented high conservation levels, emphasizing the important role played by those residues in proper enzyme activity. Therefore, both pockets were considered to assess tavaborole’s binding mode in prospective docking studies.

LeuRS is a large monomeric enzyme crucial for protein synthesis. The enzyme has two pivotal roles: aminoacylation and editing. These functions occur in distinct regions separated by about 34 Å. The aminoacylation involves the catalytic core with a typical Rossmann fold. Editing occurs in the CP1 domain, where water molecules and the 3’-OH group of tRNA play key roles. Therefore, the LeuRS’s active site accounts for the aminoacylation process whereas the CP1 editing domain accounts for the post-transfer editing of incorrectly loaded tRNAs [50]. Tavaborole’s widely accepted action mechanism indicates that this compound forms an adduct with adenosine monophosphate (AMP) (Fig 3E) within the LeuRS’s editing site; AMP works as a surrogate for 3’ terminal adenosine in tRNA^Leu^ [36].

Subsequently, molecular docking studies were conducted in both structures (SWISS-MODEL and AlphaFold) to help explain the structural basis of tavaborole’s inhibitory activity in both the CP1 domain and the active site. The SWISS-MODEL structure was employed, since it potentially captures LeuRS in a bioactive conformation where both binding sites are accessible for co-crystalized ligand of template (PDB ID: 2V0C) [36], reflecting the dynamic nature of LeuRS during its functional cycle. In contrast, AlphaFold, being a template-independent method, does not account for ligand-induced fit effects on the conformations of the active sites. Therefore, it predicts the structure without the influence of bound ligands, which may not capture the conformational changes induced by ligand binding. The major conformational differences between the LeuRS structures predicted by SWISS-MODEL and AlphaFold are highlighted in S2 Fig. Based on PH4 and rDock scores shown in Table 3, tavaborole preferentially binds in both the CP1 (-20.20 kcal/mol) and active (-24.06 kcal/mol) sites of LeuRS structure predicted by SWISS-MODEL. These findings underscore the potential of tavaborole as a potent inhibitor, with its binding affinity well-supported by the structural insights provided by the SWISS-MODEL conformation.

**Table 3:** Best docking scores among the different LeuRS domains.

Domains	rDock score (kcal/mol)	PH4 score
AlphaFold (CP1)	-7.77	0.036
SWISS-MODEL (CP1)	-20.20	0.364
AlphaFold (Active Site)	-17.0	0.211
SWISS-MODEL (Active Site)	-24.06	0.245

Furthermore, the docking protocol was validated using a set of active compounds and decoys. The enrichment rates (S3 Table) indicate that the pharmacophoric score (PH4 score) effectively ranks actives at the top of the list. Notably, tavaborole achieved the highest PH4 scores compared to a series of known actives and decoys, as highlighted by the dashed line at the top of the boxplots in S3 Fig. This robust performance underscores the reliability and accuracy of our docking protocol, which effectively differentiates between true actives and decoys. Therefore, this provides a strong foundation for predicting tavaborole’s binding affinity potential to the LeuRS structure.

Fig 3F shows tavaborole’s binding mode in LeuRS’s CP1 site (SWISS-MODEL). The primary amine of tavaborole-AMP adduct forms hydrogen bonds with amino acid residues, such as Leu336 and Tyr339, and is surrounded by hydrophobic residues, such as Met345, Ala346, and Val347. In addition, the Arg353 residue interacts with both phosphate moiety and boron atom (hydrogen bond and salt bridge, respectively). Arg353 and Thr257 also establish hydrogen bonds with other oxygen atoms, highlighting the importance of such residues in helping to inhibit protein function. Furthermore, the binding mode of tavaborole-AMP adduct in LeuRS’s active site (SWISS-MODEL) was also investigated. The adduct’s adenosine moiety interacts with Val585 and Gln582 amino acids in the active site domain through hydrogen bonds, as shown in Fig 3G. The aromatic ring near the boron atom interacts with His57 via π–π stacking interaction; Pro47 is a hydrophobic residue near the ligand. These few interactions in the active site emphasize tavaborole’s stronger interaction within the CP1 editing site. Moreover, the tavaborole’s position in the CP1 editing site showed an RMSD of 1.33 Å; this value means a quite close position in comparison to the tavaborole-*T*. *thermophilus* LeuRS complex [36] (S4 Fig). Furthermore, the ΔG binding energy was calculated for the best pose of the tavaborole-LeuRS complex, revealing a ΔG binding energy of -8.65 kcal/mol. This indicates a low energy requirement for establishing a stable interaction between tavaborole and LeuRS.

## Discussion

The versatility of antimicrobial resistance mechanisms presented by *A*. *baumannii* is a globally relevant issue since it significantly reduces the number of therapeutic options available. This factor places *A*. *baumannii* in the top priority group in WHO’s list of bacteria urgently requiring new antimicrobial drugs. Given the worrisome scenario caused by the emergence of multidrug-resistant organisms, computational strategies are an attractive and effective alternative to help screen compounds with repurposing potential and capable of controlling infections caused by the aforementioned organisms [12, 51].

The herein-adopted chemogenomic strategies enabled the tracking and identification of 31 drugs linked to 14 protein targets of *A*. *baumannii*. Among them, seven drugs that had different action mechanisms related to six different protein targets (Table 1) were prioritized to be experimentally validated against *A*. *baumannii* strains. All herein assessed targets have medicinal potential to be used as targets for new agents with antimicrobial properties. It is so because, besides being universal targets, they are involved in metabolic pathways that play an essential role in the survival, virulence, and resistance mechanisms of *A*. *baumannii*, and have already been successfully used as targets against other organisms [52–57].

MKT-077 was a candidate drug herein validated as an antimicrobial agent expected to target *A*. *baumannii* chaperone DnaK. This cationic rhodacyanine, which was originally developed as a dye, presents antiproliferative activity against cancer cell lines due to its ability to inhibit the heat shock protein 70 (Hsp70), which, in turn, belongs to the chaperone family [58]. DnaK machinery is involved in the antimicrobial resistance mechanism; thus, bacterial stress responses induced by chaperones may be used as potential targets in therapeutic alternatives [52]. According to Hosfelt et al. [59], who screened and assessed potential allosteric inhibitors of mycobacterial chaperone DnaK, telaprevir was capable of interrupting DnaK-mediated cellular proteostasis and it resulted in higher aminoglycosides-based therapy effectiveness and in reduced *Mycobacterium tuberculosis* resistance to rifampicin. MKT-077 has shown antimicrobial potential against *A*. *baumannii* in assays conducted *in vitro*. Therefore, future studies should focus on assessing its synergism with usual antimicrobials. To our knowledge, the current study was the first to identify *A*. *baumannii* DnaK as a new potential target to be used in antimicrobial therapies.

The assays conducted *in vitro* have also observed antimicrobial activity for drugs, such as atovaquone and leflunomide. Atovaquone is a hydroxynaphthoquinone clinically used to treat malaria and AIDS-associated diseases, such as pneumonia caused by *Pneumocystis carinii* and toxoplasmosis [60]. Leflunomide is an immunomodulator approved to treat rheumatoid arthritis [61]. Both drugs present the dihydroorotate dehydrogenase (DHODH) enzyme as a validated target against *A*. *baumannii*; this enzyme participates in a crucial nucleotide metabolism stage, through the *de novo* pyrimidine biosynthetic pathway, which is necessary for DNA and RNA synthesis [62]. The study conducted by Guo et al. [53] proved that defective pyrimidine biosynthesis in *Pseudomonas aeruginosa* strains accounted for reducing their virulence and antimicrobial resistance. Therefore, molecules targeting this pathway may be promising antimicrobials. Russo et al. [63] assessed a whole library of antimalarial drugs to screen potential compounds with activity in *A*. *baumannii* DHODH and identified a lead compound (DSM186) presenting MIC ≤ 1 μg/ml against the analyzed strains. This result reinforces the current findings about the potential of this target to treat infections caused by *A*. *baumannii*.

Based on studies conducted *in silico* by our research team, homoharringtonine was predicted as capable of inhibiting the 50S ribosomal subunit that plays an essential role in protein synthesis. On the other hand, this drug has shown antimicrobial potential against the assessed ATCC strain in assays conducted *in vitro*. Homoharringtonine is a plant alkaloid derived from *Cephalotaxus* trees with antitumor properties; it has been widely used to treat acute and chronic myeloid leukemia in China, since the 1970s [64]. According to Vickers et al. [56], RX-04 pyrrolocytosine—which is an antibacterial scaffold designed to bind to the bacterial 50S ribosomal subunit in a way different from that of currently used antimicrobials—was capable of inhibiting 94.7% of the assessed clinical strains, including the *A*. *baumannii* one, which recorded MIC values ranging from 1 to 4 μg/ ml. This finding highlights the importance of investigating this bacterial target to help develop specific inhibitors capable of controlling infections caused by *A*. *baumannii*, among other pathogens of great concern.

Thiabendazole, which was predicted in our studies to be a succinate dehydrogenase enzyme (SDH) inhibitor, was one of the drugs prioritized for experimental analysis showing antimicrobial potential against *A*. *baumannii*. This anthelmintic drug accounts for catalyzing succinate oxidation to fumarate and reducing the ubiquinone electron carrier to ubiquinol, for cellular energy production purposes [65]. In addition to its role in energy production processes and providing essential carbon sources for cell growth, SDH is also acknowledged for playing a pivotal role in bacterial pathogenicity [66]. According to Resch et al. [67], some tricarboxylic acid cycle genes from *Staphylococcus aureus* were upregulated in biofilms, mainly SDH genes. Therefore, given its importance for bacterial survival and virulence, SDH is a compelling target to help enhance the effectiveness of therapeutic efforts. To our knowledge, the current study was the first to identify *A*. *baumannii* SDH as a new antimicrobial target.

Ribavirin, which was another drug assessed in the current study, is a synthetic analog of the 1-β-D-ribofuranosyl-1,2,4-triazole-3-carboxamide nucleoside, which has broad antiviral activity and is currently used in combination to interferon, almost exclusively to treat infections caused by hepatitis C virus (HCV) [68]. Ribavirin acts as an inosine 5’-phosphate dehydrogenase (IMPDH) inhibitor; this enzyme is crucial to the *de novo* biosynthesis of guanine nucleotides, which, in turn, play a key role in DNA and RNA synthesis [69]. Based on the results of the current study, ribavirin is a strong *A*. *baumannii* inhibitor. Mandapati et al. [54] used a computational approach to select and assess compounds reported as *Cryptosporidium parvum* IMPDH inhibitors to investigate their antimicrobial potential against *Bacillus anthracis* IMPDH. Based on assays conducted *in vitro*, four compounds recorded MIC values ≤ 2 μM against *B*. *anthracis*, as well as presented bactericidal activity against *S*. *aureus* and *Listeria monocytogenes*. To the best of our knowledge, the current study was the first to identify *A*. *baumannii* IMPDH as a new antimicrobial target.

Tavaborole, which was predicted to be a LeuRS inhibitor, is an FDA-approved topical antifungal agent used to treat onychomycosis caused by dermatophytes [70]. Overall, aminoacyl-tRNA synthetases are a group of ubiquitous enzymes seen as attractive targets in research aimed at finding new antimicrobial agents. It is so because, besides mediating protein synthesis, they also ensure fidelity in nucleic acid information conversion into amino acids [71]. According to Li et al. [72], a compound belonging to the benzoxaborole family (GSK656), the same group to which tavaborole belongs, has shown strong inhibition power in tests conducted with *M*. *tuberculosis*. This compound has progressed to clinical development for tuberculosis treatment purposes (ClinicalTrials.gov Identifier: NCT05382312).

Tavaborole showed excellent antimicrobial activity against *A*. *baumannii* among the herein-assessed isolates, even at low concentrations; MIC values ranged from 2 to 64 μg/ml among the investigated clinical strains. Approximately 60% (9/15) of the assessed strains recorded MIC values of 2 μg/ml, including six strains with MDR profiles [45]. Moreover, all other strains recording MIC values ranging from 4 to 64 μg/ml presented MDR profiles [45]. The current results corroborate findings by Liu et al. [73], according to whom, tavaborole recorded MIC values ranging from 16 to 64 μg/ml among the assessed bacterial strains; the most promising results were observed for *Escherichia coli* (8–16 μg/ml). Results in the current study also corroborate the study by Di Bonaventura et al. [74], which recorded MIC values ranging from 7.6 to 30.2 μg/ml for the assessed MDR *P*. *aeruginosa* strains.

The present study has also confirmed tavaborole’s ability to destroy mature biofilms, as well as to reduce their metabolic activity, both in sensitive strains and in those showing some antimicrobial resistance profile, although at different levels. Tavaborole used at the concentration of 16 μg/ml was able to completely inhibit (100%) the metabolism of standard strain ATCC 19606. Metabolic activity inhibition in the assessed clinical strains reached 100% (Ab23), 93% (Ab50), 94% (Ab53), and 59% (Ab72). Results in the present study corroborate previous studies that observed increased bacterial tolerance to drugs under biofilm conditions and that required using higher antimicrobial concentrations to rule out microorganisms. This behavior may be associated with the protective properties of persistent cells found in biofilms, as well as with inadequate antimicrobial agent penetration into the extracellular polymeric matrix [75, 76].

Previous studies focused on assessing the effect of conventional antimicrobials on planktonic cells and those incorporated into *A*. *baumannii* biofilms [77, 78], recorded a 4- to 50-fold increase in the concentration required to kill biofilm-incorporated cells. However, these values can be even higher, i.e., can reach up to more than 1,000 [79] or 10,000 [77] times their respective MIC values. Antimicrobial drug concentrations capable of biofilm inhibition, in the aforementioned studies, were so higher than the respective MIC values observed for the planktonic condition that their clinical use was deemed unfeasible. Although the antimicrobial drug concentration herein capable of destroying cells under biofilm condition (16 μg/ml) was higher than the observed MIC value (2 μg/ml)—which is expected due to biofilm behavior -, this value was not that high and it was within the MIC range described by Liu et al. [73] and Di Bonaventura et al. [74] for the assessed planktonic forms of *A*. *baumannii*.

Our findings strongly suggest that tavaborole stands out as a promising antimicrobial candidate for pre-clinical studies. Contrary to the historical association of boron-containing compounds with high toxicity in insects, often exploited for their pesticidal properties, tavaborole exhibits an exceptional safety profile. In preclinical safety assessments, the tavaborole demonstrated remarkable attributes. Specifically, even at a concentration of 10 μM, tavaborole did not exert inhibitory effects on key cytochrome P450 isoforms, including CYP1A2, CYP2C9, CYP2C19, CYP2D6, or CYP3A4 [80]. The carcinogenicity assessment of tavaborole through a conventional 2-year study in mice and rats, involving oral doses ranging from 12.5 to 50 mg/kg/day, did not reveal any drug-related neoplastic findings, highlighting its safety profile. Furthermore, tavaborole exhibited no mutagenic or clastogenic potential based on comprehensive *in vitro* genotoxicity assessments, including the Ames assay and Human lymphocyte chromosomal aberration assay, as well as an *in vivo* genotoxicity test, the rat micronucleus assay [81]. Notably, fertility evaluations in male and female rats receiving oral doses up to 300 mg/kg/day demonstrated no adverse effects, underscoring the compound’s reproductive safety. In an embryofetal development context, a meticulous examination in rats and rabbits at varying dose levels illustrated specific findings associated with maternal toxicity, such as embryofetal resorption and skeletal malformations at the highest dose of 300 mg/kg/day in rats. However, at lower doses, including 100 mg/kg/day in rats and 150 mg/kg/day in rabbits, no developmental toxicity or drug-related malformations were observed. The absence of embryofetal mortality in rabbits at 50 mg/kg/day further supports tavaborole’s favorable safety profile. Furthermore, an oral pre- and post-natal development study in rats, utilizing doses up to 100 mg/kg/day, demonstrated no adverse effects on embryofetal development or post-natal outcomes, even in the presence of minimal maternal toxicity [81].

Additionally, previous studies have investigated and determined that tavaborole does not show cytotoxicity in bronchial epithelial cells (concentrations until 0.8 mM) [74], nor neurotoxicity or nephrotoxicity potential in several human normal and cancer cell lines [73], besides being considered non-carcinogenic and non-mutagenic [82, 83]. Collectively, these comprehensive toxicological investigations, along with the antimicrobial effects of benzoxaborole compounds on other organisms [36, 55, 72, 73, 84–87], provide robust evidence supporting the safety of tavaborole, corroborate our current findings, and reinforce its potential for prospective in vivo repurposing studies.

Given the antimicrobial and antibiofilm potential presented by tavaborole, the structural bases of its activity were investigated through molecular docking studies. Docking results have indicated that the LeuRS’s active site, in both predicted 3D structures (SWISS-MODEL and AlphaFold), was big enough to receive several ligands. However, because cross-docking assesses the ligand’s position compared to the template’s position, the PH4 score is more impactful than the binding score (rDock score), since it is based on overlapping features. The docking protocol was validated by testing its ability to rank known active compounds better than inactive decoys. Notably, tavaborole, a known inhibitor, received the highest score, further confirming the accuracy of the docking protocol in distinguishing active molecules from inactive ones. The results (S3 Table and S3 Fig) demonstrate that the scoring system (PH4 score) successfully prioritizes active compounds. Thus, the herein-performed docking analysis indicated that tavaborole’s structure binds very similarly to the template (PDB ID: 2V0C) [36] of the CP1 domain generated by the homology model (SWISS-MODEL). This finding enables classifying the SWISS-MODEL LeuRS 3D structure as the one most likely to interact with tavaborole in the CP1 editing site, besides indicating the right pocket where tavaborole interacts. Moreover, the MM/GBSA analysis revealed a binding interaction between tavaborole and LeuRS. Altogether, the current molecular docking results enabled rationalizing interactions between the tavaborole-AMP complex and the LeuRS editing site, and it corroborated results observed by Rock et al. [36], according to whom, the compound binds to the 3′-terminal of the tRNA^Leu^, forms a complex in the LeuRS editing domain and blocks the catalytic function of this enzyme. These key features may be used as a structural basis to design new tavaborole analogs with improved CP1-LeuRS inhibiting effects.

Based on results in the current study, the herein adopted computational approach, which was validated by experimental methods, effectively enabled screening and identifying seven drugs with potential antimicrobial activity against *A*. *baumannii*. Some of the repurposed drugs have shown strong inhibitory potential. Tavaborole was the most promising candidate among them; it recorded a MIC value of 2 μg/ml, as well as antibiofilm activity at 16 μg/ml, for both the standard ATCC strain and the assessed clinical isolates. LeuRS, which was the target predicted in the current study, is an essential enzyme not only for microbial growth but also for proteostasis maintenance. The interruption of metabolic events controlled by it may not only interrupt infectious processes by inhibiting bacterial proliferation but also suppress β-lactamase production, which is one of the most worrisome carbapenem-resistance mechanisms since these antimicrobials are among the last resorts to treat MDR infections. Moreover, tavaborole may provide a promising opportunity to treat nosocomial infections caused by *A*. *baumannii* MDR/XDR/PDR, since it attacks a target type different from those conventionally targeted by currently used antimicrobials, besides having great potential to be used in clinical applications.

The present study has some limitations. Selection criteria herein prioritized for drug screening purposes do not allow ruling out the possibility that some drugs that were not herein assessed may also have repurposing potential to treat infections caused by *A*. *baumannii*. Although molecular dynamics simulations were not performed in this study, the conclusions presented are not compromised because they were based on X-ray structures of the tavaborole-LeuRS complex from other organisms available in the literature, together with the presented molecular docking studies that supported the binding mode and probable inhibition mechanism of tavaborole. Finally, because no tests were carried out with animal models, it is necessary to conduct additional studies to prove the therapeutic advantages of the investigated drugs through assays *in vivo*.

## Conclusion

The strategies adopted in this study revealed seven drugs with different action mechanisms that showed potential antimicrobial activity against *A*. *baumannii*. Among them, tavaborole was the most promising antimicrobial; it recorded a MIC value of 2 μg/ml and strong antibiofilm activity (16 μg/ml) against both the standard ATCC strain and MDR clinical strains. Furthermore, our docking studies have indicated interactions between the tavaborole-AMP complex and the LeuRS editing site (CP1 domain).

## Supporting information

S1 Fig Minimum bactericidal concentration (MBC) assay applied to MKT-077. Layout of MBC assay plates used to test MKT-077’s antimicrobial potential against the ATCC strain, at drug concentrations ranging from 1,024 to 256 μg/ml. Each plate represents a drug concentration test (1,024, 512, and 256 μg/ml); different serial dilutions (10^−3^ to 10^−6^) were spotted on agar in each column of the plate, in triplicates. Plate C+: growth control—bacteria incubated without any drug were serially diluted and plated.
(TIF)

S2 Fig Conformational differences of *Acinetobacter baumannii* LeuRS generated by SWISS-MODEL (cyan) and AlphaFold (gray). (a) Illustrates the conformational differences in the CP1 editing domain, highlighting structural variations between the two models, and (b) shows the differences in the active site, emphasizing the distinct conformations predicted by SWISS-MODEL and AlphaFold.
(TIF)

S3 Fig Boxplot analysis of docking enrichment. (A) Shows the docking validation of the editing site, while (B) presents the validation of the active site. The figure utilizes the PH4 scores for the evaluation. The dashed line marks the score of the tavaborole.
(TIF)

S4 Fig Superimposed representation of redocked tavaborole (yellow) and co-crystallized template (green) in CP1 editing site (RMSD: 1.33 Å). (TIF)

S1 Table Pharmacokinetic parameters of drugs evaluated for repurposing. Note: **Pharmacokinetic properties:** obtained from the DrugBank [22], TTD [21], and PubChem [28]. **T**_**max**_**:** time to reach maximum plasma concentration. *Total clearance estimated: considering an average body weight of 70 kg by the following equations: Cl_T_ = V_d._ k_e_, where k_e_ = 0,693/t_1/2_ (Cl_T_ = total clearance; V_d_ = volume of distribution; k_e_ = elimination rate constant; t_1/2_: half-life).
(DOCX)

S2 Table Geometric quality characteristics of LeuRS 3D model developed using SWISS-MODEL, AlphaFold, and I-TASSER. (DOCX)

S3 Table AUC, EF, and BEDROC values from docking of actives and decoys into the CP1 editing and active sites. (DOCX)

S1 File 3D structure of LeuRS from *Acinetobacter baumannii* generated by AlphaFold in. pdb format. (PDF)

S2 File 3D structure of LeuRS from *Acinetobacter baumannii* generated by SWISS-MODEL in. pdb format. (PDF)

S3 File Supplementary methods. (DOCX)

S1 Raw data The file “Raw data.xlsx” contains all data obtained and used to generate Figs 1 and 2 and S3 Fig. A more detailed explanation is presented in the first spreadsheet of this excel file.
(XLSX)
