# Supplementary figures and images for: New antibacterial candidates against Acinetobacter baumannii discovered by in silico-driven chemogenomics repurposing
Source: PLoS One. 2024 Sep 26;19(9):e0307913. doi: 10.1371/journal.pone.0307913 (PMC11426455; doi:10.1371/journal.pone.0307913)

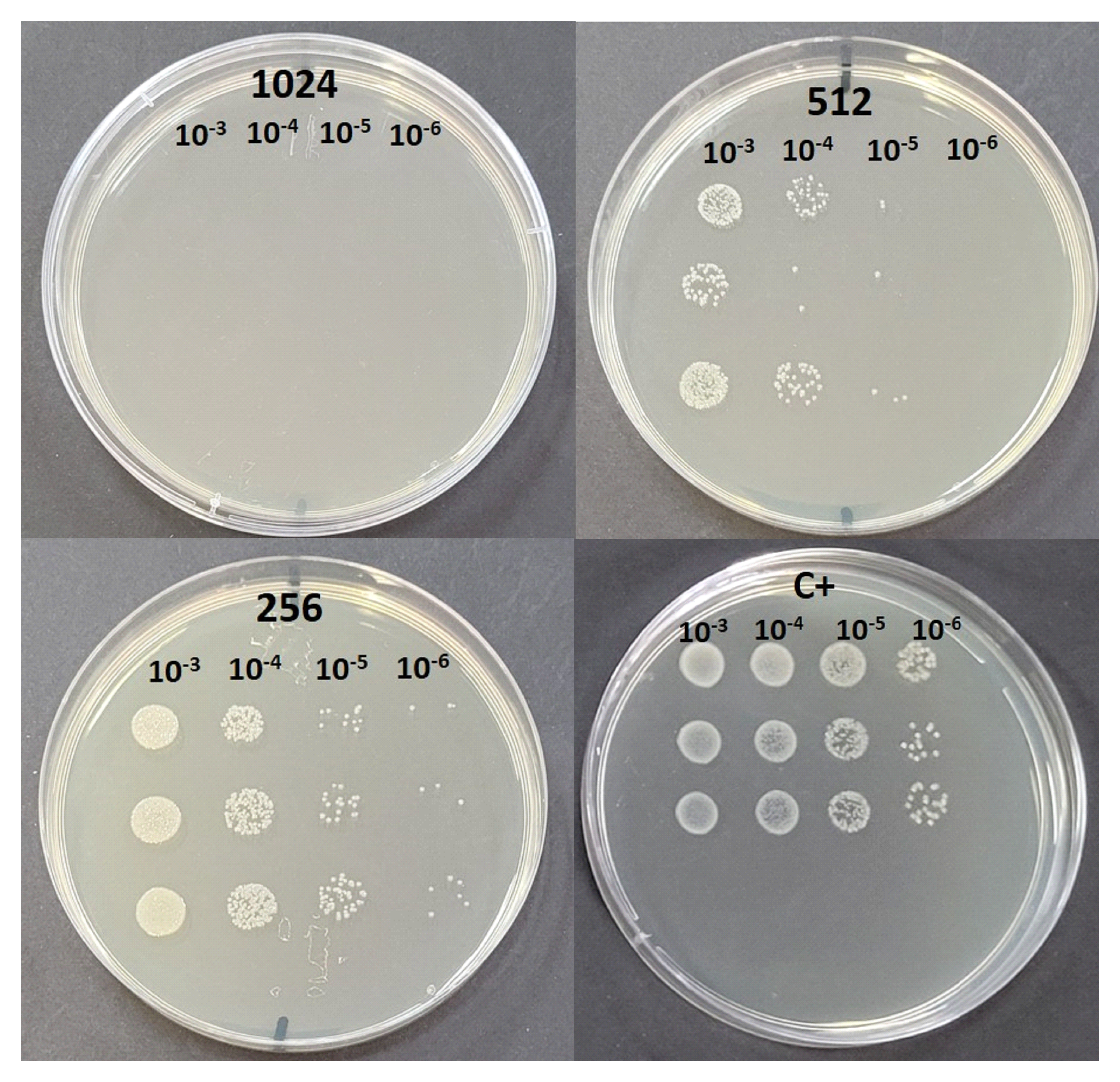

Supplement: S1 Fig — Layout of MBC assay plates used to test MKT-077’s antimicrobial potential against the ATCC strain, at drug concentrations ranging from 1,024 to 256 μg/ml. Each plate represents a drug concentration test (1,024, 512, and 256 μg/ml); different serial dilutions (10−3 to 10−6) were spotted on agar in each column of the plate, in triplicates. Plate C+: growth control—bacteria incubated without any drug were serially diluted and plated. (TIF) [file pone.0307913.s001.tif]

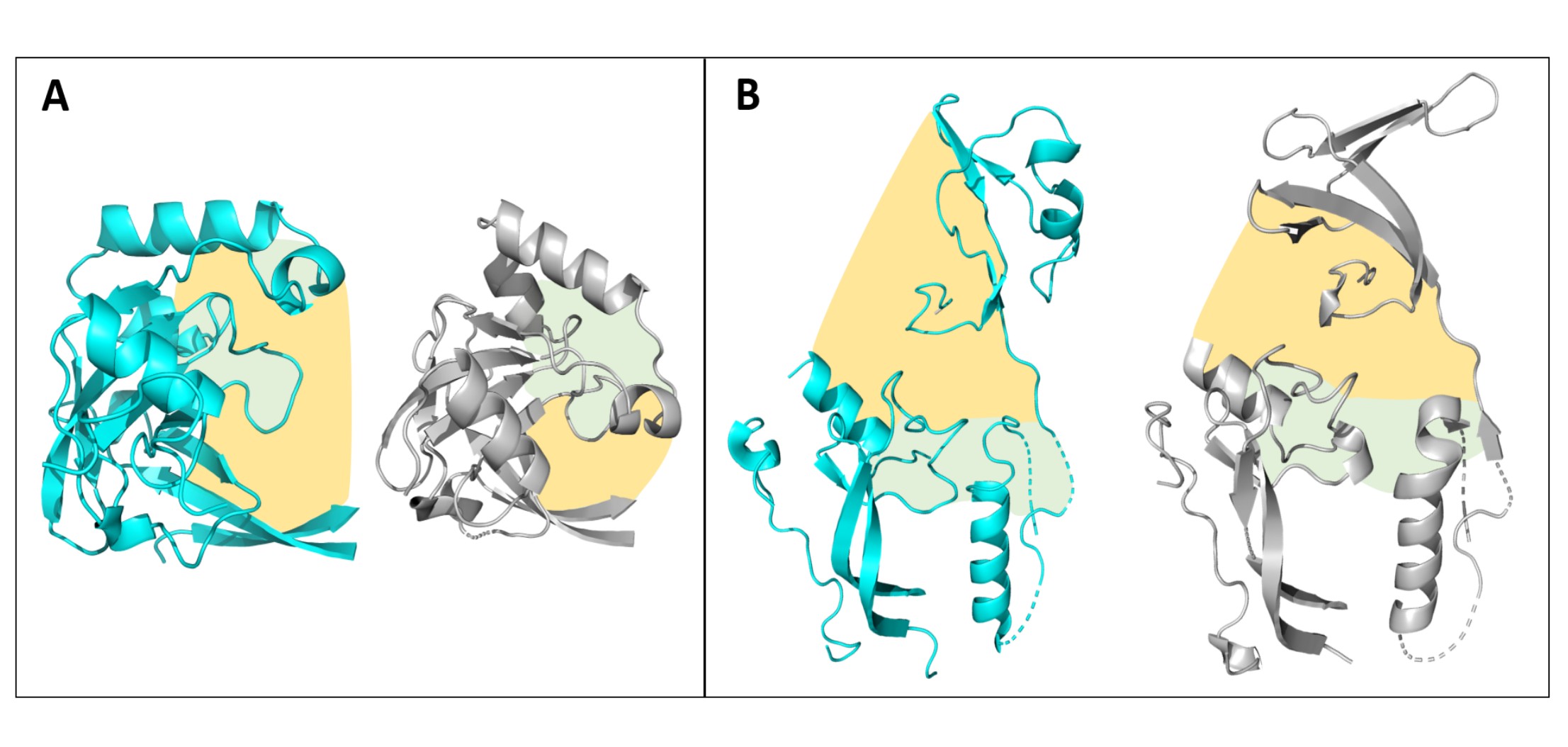

Supplement: S2 Fig — (a) Illustrates the conformational differences in the CP1 editing domain, highlighting structural variations between the two models, and (b) shows the differences in the active site, emphasizing the distinct conformations predicted by SWISS-MODEL and AlphaFold. (TIF) [file pone.0307913.s002.tif]

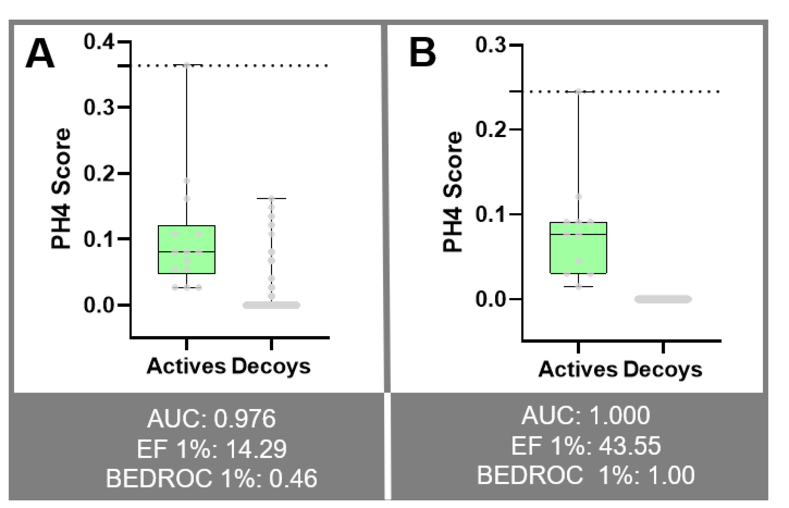

Supplement: S3 Fig — (A) Shows the docking validation of the editing site, while (B) presents the validation of the active site. The figure utilizes the PH4 scores for the evaluation. The dashed line marks the score of the tavaborole. (TIF) [file pone.0307913.s003.tif]

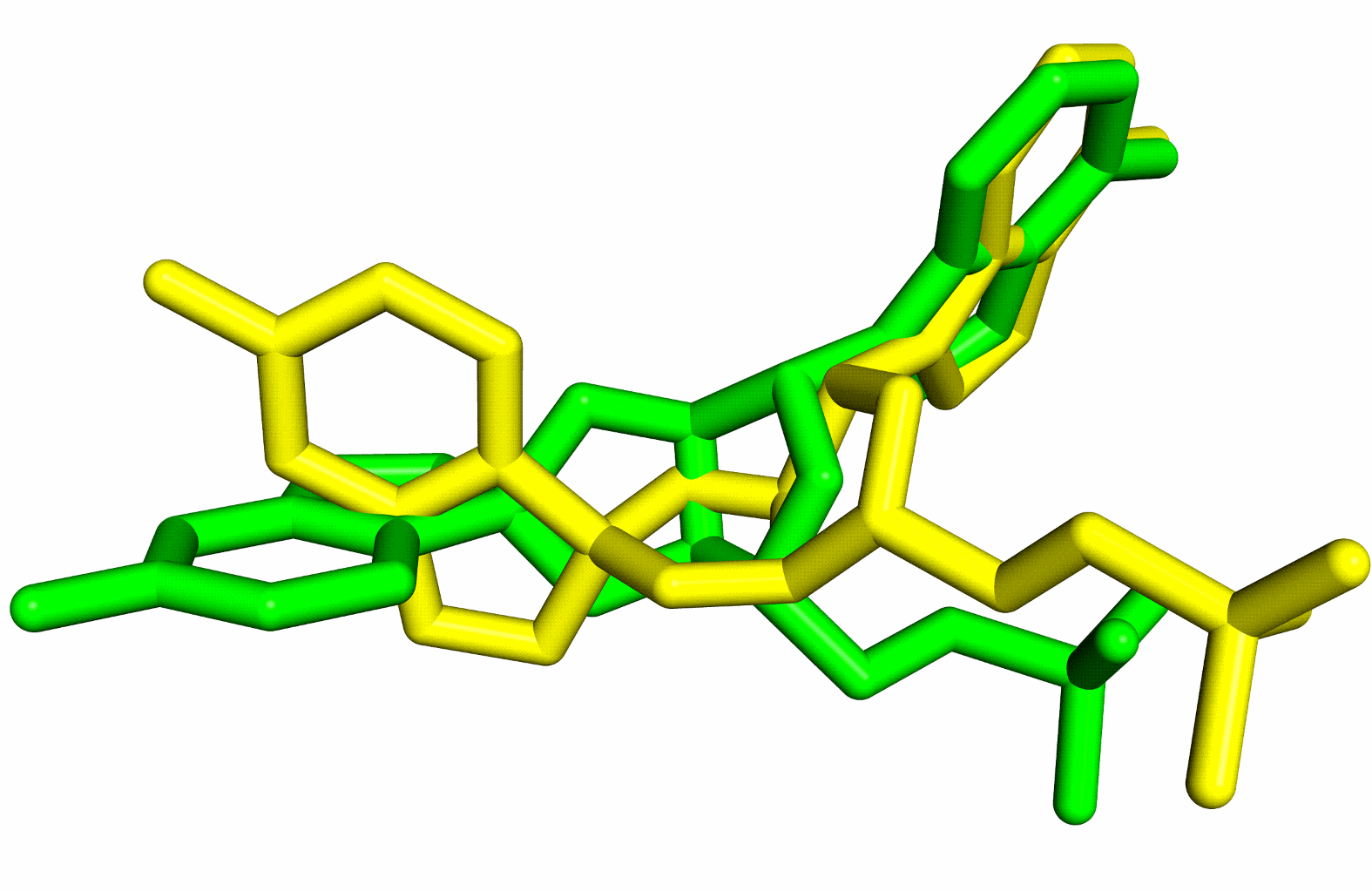

Supplement: S4 Fig — (TIF) [file pone.0307913.s004.tif]
