# Supplementary material for: New antibacterial candidates against Acinetobacter baumannii discovered by in silico-driven chemogenomics repurposing
Source: PLoS One. 2024 Sep 26;19(9):e0307913. doi: 10.1371/journal.pone.0307913 (PMC11426455; doi:10.1371/journal.pone.0307913)
Supplement: S1 Table — Note: Pharmacokinetic properties: obtained from the DrugBank [22], TTD [21], and PubChem [28]. Tmax: time to reach maximum plasma concentration. *Total clearance estimated: considering an average body weight of 70 kg by the following equations: ClT = Vd. ke, where ke = 0,693/t1/2 (ClT = total clearance; Vd = volume of distribution; ke = elimination rate constant; t1/2: half-life). (DOCX) [file pone.0307913.s005.docx]

**S1 Table. Pharmacokinetic parameters of drugs evaluated for repurposing.**

| DRUG | GROUP | BIOAVAILABILITY | VOLUME OF DISTRIBUTION | PROTEIN BINDING | EXCRETION ROUTES | HALF-LIFE | CLEARANCE | LOG P | MOLECULAR WEIGHT | TOXICITY (LD50) |  |
| --- | --- | --- | --- | --- | --- | --- | --- | --- | --- | --- | --- |
| Atovaquone | Approved | Oral bioavailability: 47% (with food) and 23% (without food) | 0,60 ± 0,17 L/kg | Not Available | Enterohepatic cycling and eventual fecal elimination | 2.2 - 3.2 days | 0.624 L/h* | 5.8 | 366.8 g/mol | 0.05 mol/kg  (mice and rat) |  |
| Homoharringtonine | Approved | Not Available | 141 ± 93.4 L/kg | ≤ 50% | The main route of elimination is unknown. Renal elimination  ≤ 15% | ≈ 6 hours | 1140 L/h* | 2.09 | 545.6 g/mol | 3.15 mol/kg (rat) |  |
| Leflunomide | Approved | T_max_: 6-12 hours (oral administration) | 0.13 L/kg | > 99.3% | 43% (urine) and 48% (feces) | 2 weeks | 0,0188 L/h* | 2.8 | 270.21 g/mol | 3.03 mol/kg (rat) |  |
| MKT-077 | Clinical trial - Phase I | T_max_: 1 min after injection (intravenous administration) | 6.8 L/kg | Not Available | 33.5% (urine) and 61.1% (feces) | 1.8–4 hours | 126 L/h* | 3.36 | 432 g/mol | 0.000116 mol/kg (mouse) |  |
| Ribavirin | Approved | T_max_: 2 hours  Oral bioavailability: 64% | Large volume of distribution | No protein binding reported | 61% (urine) and 12% (feces) | 120 -170 hours | 26 L/h* | - 1.9 | 244.2 g/mol | 0.011 mol/kg (rat) |  |
| Tavaborole | Approved | 5.9 ± 4.9 ng/ml (topical application) | Not Available | Not Available | Primarily renal | 28.5 hours | Not Available | 1.51 | 151.93 g/mol | >0.0033 mol/kg (rat) |  |
| Thiabendazole | Approved | T_max_: 1 - 2 hours (oral administration) | Not Available | Not Available | Renal elimination | 0.9 - 2 hours | Not Available | 2.47 | 201.25 g/mol | 2.02 mol/kg rat) | |
| Note:  Pharmacokinetic properties: obtained from the DrugBank [4], TTD [5], and PubChem [6].  T_max_: time to reach maximum plasma concentration.  *Total clearance estimated: considering an average body weight of 70 kg by the following equations: Cl_T_ = V_d_ . k_e_, where k_e_ = 0,693/t_1/2_ (Cl_T_ = total clearance; V_d_ = volume of distribution; k_e_ = elimination rate constant; t_1/2_: half-life). | | | | | | | | | | | |
