# Supplementary material for: New antibacterial candidates against Acinetobacter baumannii discovered by in silico-driven chemogenomics repurposing
Source: PLoS One. 2024 Sep 26;19(9):e0307913. doi: 10.1371/journal.pone.0307913 (PMC11426455; doi:10.1371/journal.pone.0307913)
Supplement: S2 Table — (DOCX) [file pone.0307913.s006.docx]

**S2 Table. Geometric quality characteristics of LeuRS 3D model developed using SWISS-MODEL, AlphaFold, and I-TASSER.**

| **Parameters** | **SWISS-MODEL Score** | **AlphaFold Score** | **I-TASSER Score** |
| --- | --- | --- | --- |
| Clashscore | 1.83 | 0.95 | 3.29 |
| MolProbity score | 1.32 | 0.87 | 2.50 |
| Poor rotamers | 7 (1.04%) | 2 (0.87%) | 8 (10.93%) |
| Ramachandran Outliers | 6 (0.75%) | 3 (0.34%) | 32 (3.67%) |
| Ramachandran Favored | 762 (94.78%) | 851 (97.59%) | 771 (88.42%) |
| Bad Angles | 67 (0.75%) | 24 (0.25%) | 91 (0.95%) |
| Bad Bonds | 3 (0.05%) | 0 (0.0%) | 0 (0.0%) |
