# Supplementary material for: New antibacterial candidates against Acinetobacter baumannii discovered by in silico-driven chemogenomics repurposing
Source: PLoS One. 2024 Sep 26;19(9):e0307913. doi: 10.1371/journal.pone.0307913 (PMC11426455; doi:10.1371/journal.pone.0307913)
Supplement: S3 Table — (DOCX) [file pone.0307913.s007.docx]

**S3 Table. AUC, EF, and BEDROC values from docking of actives and decoys into CP1 editing and active sites.**

| **Enrichment analysis** | **Binding site** | **AUC** | **EF 1%** | **EF 5%** | **EF 10%** | **BEDROC 1%** | **BEDROC 5%** | **BEDROC 10%** |
| --- | --- | --- | --- | --- | --- | --- | --- | --- |
| rDock score | CP1 site | 0.720 | 7.14 | 5.71 | 4.29 | 0.21 | 0.23 | 0.32 |
| rDock score | Active site | 0.456 | 0.00 | 0.00 | 0.00 | 0.00 | 0.00 | 0.01 |
| PH4 score | CP1 site | 0.976 | 14.29 | 11.57 | 1.00 | 0.46 | 0.55 | 0.70 |
| PH4 score | Active site | 1.000 | 43.55 | 1.00 | 1.00 | 1.00 | 1.00 | 1.00 |
