# Supplementary material for: New antibacterial candidates against Acinetobacter baumannii discovered by in silico-driven chemogenomics repurposing
Source: PLoS One. 2024 Sep 26;19(9):e0307913. doi: 10.1371/journal.pone.0307913 (PMC11426455; doi:10.1371/journal.pone.0307913)
Supplement: S3 File — (DOCX) [file pone.0307913.s010.docx]

**Supplementary Methods**

**Enrichment analysis**

A series of 14 LeuRS inhibitors with IC_50_ ≤ 1 μM were retrieved from the literature [1]. Then, 468 decoys were generated from the LIDEB’s Useful Decoys webapp [2]. Decoys were chosen if they were similar to active compounds according to five physicochemical descriptors (cLogP, number of rotational bonds, number of hydrogen bond donors and acceptors, and molecular weight) and structurally different from the active compounds (Tanimoto coefficient ≤ 0.20).

The enrichment rates of the docking models were calculated using Area Under the ROC Curve (AUC), Enrichment Factor (EF), and Boltzmann-Enhanced Discrimination of ROC (BEDROC) [3]. The AUC and EF were calculated as follows:

$AUC = \frac{1}{n} \sum_{i=1}^{n} \left( 1- f_{1} \right)$ (Equation 1)

$\mathrm{EF}_{x\%}= \frac{n_{x\%}/N_{x\%}}{n / N}$ (Equation 2)

where, $f_{1}$ is the fraction of decoys ranked higher than the *i*th active, *n* represents the total number of actives, *N* represents the total number of compounds (actives and decoys) in the benchmarking dataset, whereas $n_{x\%}$ and $N_{x\%}$ represents the number of actives and represents the total number of compounds in the *x*% ordered list, respectively. Although extremely important, the AUC and EF are not able to discern the order of actives and decoys in the top x% list [3]. To overpower the “early recognition” issue, BEDROC was calculated as follows:

$RIE= \frac{\frac{1}{n}\sum_{i=n}^{n} e^{-{\alpha x}_{i}}}{\frac{1}{n} \left( \frac{{1-e}^{-\alpha}}{e\frac{\alpha}{n}-1} \right)}$ (Equation 3)

$BEDROC=RIE x \frac{R_{a}\sinh\left( \frac{\alpha}{2} \right)}{\cosh\left( \frac{\alpha}{2} \right)-cosh\left( \frac{\alpha}{2}- \alpha R_{\alpha} \right)}+ \frac{1}{1-exp\left( \alpha\left( 1-R_{\alpha} \right) \right)}$ (Equation 4)

where $x_{i}$ is the relative rank of the *i*th active, α is an exponential weighting factor that controls the emphasis given to early recognition, and $R_{\alpha}$ is the proportion of actives in the benchmarking set.

**Supplementary References**

1. Palencia A, Li X, Bu W, Choi W, Ding CZ, Easom EE, et al. Discovery of Novel Oral Protein Synthesis Inhibitors of *Mycobacterium tuberculosis* That Target Leucyl-tRNA Synthetase. Antimicrob Agents Chemother. 2016;60(10):6271–80. https://journals.asm.org/doi/10.1128/AAC.01339-16

2. Gori DNP, Alberca LN, Rodriguez S, Alice JI, Llanos MA, Bellera CL, et al. LIDeB Tools: A Latin American resource of freely available, open-source cheminformatics apps. Artif Intell Life Sci. 2022;2:100049. https://linkinghub.elsevier.com/retrieve/pii/S2667318522000198

3. Neves BJ, Mottin M, Moreira-Filho JT, Sousa BK de P, Mendonca SS, Andrade CH. Best Practices for Docking-Based Virtual Screening. In: Molecular Docking for Computer-Aided Drug Design. Elsevier; 2021. p. 75–98. https://linkinghub.elsevier.com/retrieve/pii/B9780128223123000011
